# Targeted promoter editing for rice resistance to *Xanthomonas oryzae* pv. *oryzae* reveals differential activities for *SWEET14*‐inducing TAL effectors

**DOI:** 10.1111/pbi.12613

**Published:** 2016-12-17

**Authors:** Servane Blanvillain‐Baufumé, Maik Reschke, Montserrat Solé, Florence Auguy, Hinda Doucoure, Boris Szurek, Donaldo Meynard, Murielle Portefaix, Sébastien Cunnac, Emmanuel Guiderdoni, Jens Boch, Ralf Koebnik

**Affiliations:** ^1^ UMR Interactions Plantes Microorganismes Environnement (IPME)IRD‐CIRAD‐UniversitéMontpellierFrance; ^2^ Institut für BiologieInstitutsbereich GenetikMartin‐Luther‐Universität Halle‐WittenbergHalle (Saale)Germany; ^3^ CIRADUMR AGAP (Amélioration génétique et Adaptation des Plantes)MontpellierFrance; ^4^ Present address: LabEx CeMEBUniversité de MontpellierMontpellierFrance; ^5^ Present address: Institut für PflanzengenetikLeibniz Universität HannoverHannoverGermany; ^6^ Present address: Sustainable Agro Solutions S.A.Almacelles (Lleida)Spain

**Keywords:** bacterial leaf blight, susceptibility gene, genome engineering, TALEN, transgene‐free plants

## Abstract

As a key virulence strategy to cause bacterial leaf blight, *Xanthomonas oryzae* pv. *oryzae* (*Xoo*) injects into the plant cell DNA‐binding proteins called transcription activator‐like effectors (TALEs) that bind to effector‐binding elements (EBEs) in a sequence‐specific manner, resulting in host gene induction. TALEs AvrXa7, PthXo3, TalC and Tal5, found in geographically distant *Xoo* strains, all target *OsSWEET14*, thus considered as a pivotal TALE target acting as major susceptibility factor during rice–*Xoo* interactions. Here, we report the generation of an allele library of the *OsSWEET14* promoter through stable expression of TALE‐nuclease (TALEN) constructs in rice. The susceptibility level of lines carrying mutations in AvrXa7, Tal5 or TalC EBEs was assessed. Plants edited in AvrXa7 or Tal5 EBEs were resistant to bacterial strains relying on the corresponding TALE. Surprisingly, although indels within TalC EBE prevented *OsSWEET14* induction in response to BAI3 wild‐type bacteria relying on TalC, loss of TalC responsiveness failed to confer resistance to this strain. The TalC EBE mutant line was, however, resistant to a strain expressing an artificial *SWEET14*‐inducing TALE whose EBE was also edited in this line. This work offers the first set of alleles edited in TalC EBE and uncovers a distinct, broader range of activities for TalC compared to AvrXa7 or Tal5. We propose the existence of additional targets for TalC beyond *SWEET14*, suggesting that TALE‐mediated plant susceptibility may result from induction of several, genetically redundant, host susceptibility genes by a single effector.

## Introduction

Preventing colonization by pathogenic microorganisms is one of the major challenges for plants during development. Classically, plant resistance traits are governed by the so‐called dominant *R* genes that typically encode nucleotide‐binding leucine‐rich repeat (NB‐LRRs) proteins, which detect the molecular activity of pathogen effector proteins in the plant cell (Cui *et al*., 2015). Alternatively, recessive immunity to adapted pathogenic microbes can emerge from the mutation, or the loss, of a susceptibility (*S*) gene that acts as a basic host–pathogen compatibility factor to promote disease. In breeding for resistance, altering *S* genes to counteract the infection strategy represents an interesting and potentially more durable alternative to the introduction of dominant *R* genes (van Schie and Takken, 2014).

Bacterial leaf blight (BLB) is a widespread vascular rice disease caused by *Xanthomonas oryzae* pv. *oryzae* (*Xoo*), which severely reduces grain yield and represents a major threat for global food security. In Asia, BLB control strategies rely essentially on genetic resistance. African BLB pathogens were found to be genetically distinct from Asian isolates, and effective rice resistances against African isolates have not yet been deployed (Gonzalez *et al*., 2007; Poulin *et al*., 2015; Verdier *et al*., 2012). *Xoo* pathogenicity depends on a specific class of virulence factors, called TALEs (transcription activator‐like effectors), which resemble eukaryotic transcriptional activators (for review, see Hutin *et al*., 2015a). Upon translocation into the plant cell and import in the nucleus, TALEs bind to specific promoter elements (effector‐binding elements, EBEs) following a DNA recognition code where the repeat‐variable diresidues (RVDs) of each repeat forming the TALE DNA‐binding domain interact with a specific nucleotide (Boch *et al*., 2009; Moscou and Bogdanove, 2009). This recognition initiates transcription of the targeted gene, whose function often determines the outcome of the interaction.

Abundant genetic data suggest that rice resistance mechanisms to *Xoo* exhibit atypical features (Zhang and Wang, 2013). The rice genome encodes over 400 NB‐LRRs proteins but only one of them (*Xa1*) has been shown to confer resistance to a few *Xoo* strains (Yoshimura *et al*., 1998). Instead, rice resistance to *Xoo* often relies on executor (*E*) genes distinct from classical *R* genes, whose transcriptional activation by TALEs triggers immunity, leading to dominant resistance (for review, see Zhang *et al*., 2015). Alternatively, resistance can be conferred by recessive alleles corresponding to mutated forms of susceptibility genes (for review, see Iyer‐Pascuzzi and McCouch, 2007; Kottapalli *et al*., 2007) and results in this case from the loss of induction of a gene essential to disease (Hutin *et al*., 2015a). This type of resistance alleles includes promoter‐mutated forms of the nodulin *MtN3*/*SWEET* gene family, occurring in the rice natural diversity, which function as TALE‐unresponsive resistance alleles against Asian *Xoo* strains due to DNA polymorphism in the EBEs recognized by the cognate TALEs. For example, it was shown that the recessive *xa13* resistance allele was derived from a mutation in the promoter region of *Os8N3*/*SWEET11* recognized by the TALE PthXo1 from the Philippine strain PXO99^A^ (Chu *et al*., 2006). Similarly, whereas PthXo2 from *Xoo* strain JXO1^A^ (Japan) drives *Os12N3*/*SWEET13*/*Xa25* expression in the susceptible *indica* rice variety IR24 through direct binding to a 22‐bp EBE, *japonica* varieties (including Nipponbare) that are resistant to *Xoo* bacteria relying on PthXo2 display a single‐nucleotide polymorphism (SNP) at the 4th position of the PthXo2 EBE within the *SWEET13* promoter, thus preventing its induction upon infection (Richter *et al*., 2014; Zhou *et al*., 2015). As they govern situations of recessive resistance or susceptibility (Hutin *et al*., 2015a; Zhang *et al*., 2015), polymorphic promoter sequences of *SWEET* genes can be of special interest for resistance engineering strategies.

*SWEET11*,* SWEET13* and *SWEET14*, belonging to *SWEET* family clade III, have been shown to be targeted by several TALEs (Antony *et al*., 2010; Yang *et al*., 2006; Zhou *et al*., 2015), and systematic analysis of rice *SWEET* paralogs further revealed that all, and only, clade‐III members can act as susceptibility genes (Streubel *et al*., 2013). Because they encode sugar transporters mediating glucose and sucrose export, *SWEET* gene induction by TALEs is thought to trigger sugar release to the apoplast, providing a nutrient source to the pathogen (Chen, 2014; Chen *et al*., 2015; Cohn *et al*., 2014).

*Os11N3*/*SWEET14* stands out as an interesting example of convergent evolution because it is targeted by unrelated TALEs from multiple, phylogenetically distinct *Xoo* strains: AvrXa7 from strain PXO86 (Philippines), PthXo3 from strain PXO61 (Philippines), Tal5 from strain MAI1 (Mali) and TalC from strain BAI3 (Burkina Faso) (Antony *et al*., 2010; Chu *et al*., 2006; Streubel *et al*., 2013; Yu *et al*., 2011; Zhou *et al*., 2015). Interestingly, EBEs recognized by these four TALEs were found to overlap or to be in a close vicinity (Hutin *et al*., 2015a). In particular, TalC directly activates *SWEET14* through recognition of a DNA box located upstream from the AvrXa7, PthXo3 and Tal5 EBEs (Yu *et al*., 2011). Engineering mutations within AvrXa7 EBE in the *Os11N3*/*SWEET14* promoter resulted in disease resistance against an Asian *Xoo* strain carrying the AvrXa7 effector (Li *et al*., 2012). In addition, a naturally occurring deletion encompassing AvrXa7 and Tal5 EBEs in the *Oryza barthii* wild rice species was recently shown to confer broad‐spectrum resistance to bacterial blight (Hutin *et al*., 2015b). These data support the current view that major virulence TALEs target a single major susceptibility gene.

The TalC effector from African *Xoo* strain BAI3 has been identified in a mutant screen for loss of virulence on susceptible rice varieties (Yu *et al*., 2011). As a *talC* mutant is severely affected in virulence and *talC trans*‐complementation restores virulence, TalC is considered as BAI3's major virulence TALE. However, because no mutation in TalC EBE has been engineered nor identified in the rice natural diversity so far, data are still lacking to formally attest that TalC virulence activity solely consists in *SWEET14* induction. To address this question and to generate sources of resistance to *Xoo* African strains relying on TalC, we have deployed a genome editing approach based on TALE‐nucleases (TALENs). TALENs are fusions between designer TALE modules with customized recognition specificity and the nuclease domain of the type IIS restriction enzyme *Fok*I (Chen and Gao, 2013; Li *et al*., 2011; Sun and Zhao, 2013). Target site recognition and TALEN dimerization triggers a double‐strand break (DSB), which in turn induces non‐homologous end joining (NHEJ)‐mediated DNA repair pathways and generates small random insertions or deletions at the cleavage site, resulting in an ‘edited’ sequence.

To compare the relative contributions of multiple EBEs within the *SWEET14* promoter, we targeted the AvrXa7, Tal5 and TalC EBEs for mutagenesis. Expression and pathogenicity assays revealed that disruption of AvrXa7‐ and Tal5‐mediated *SWEET14* induction rendered edited rice plants resistant to *Xoo* infection. Surprisingly, modifications of the TalC EBE failed to confer resistance to bacteria relying on TalC for infection, thus suggesting that this major virulence TALE can mediate plant disease through induction of more than a single susceptibility gene.

## Results

### Generation of TALEN‐expressing transgenic rice and selection of lines edited in AvrXa7, Tal5 and TalC EBEs

**Figure 1:**
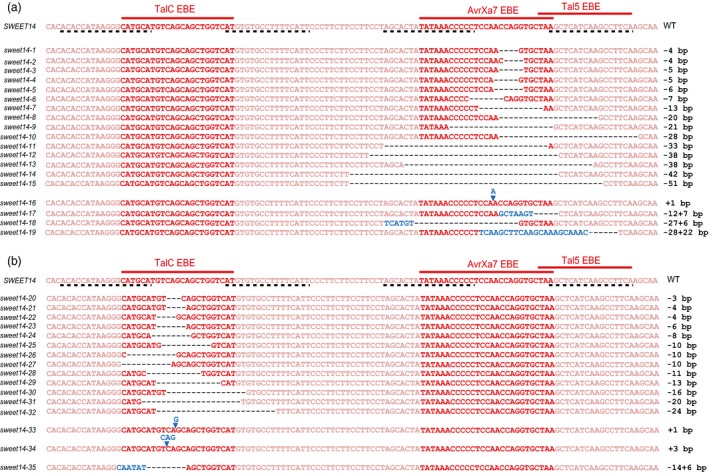
Alignment of a *SWEET14* promoter fragment in selected rice (cv. Kitaake) lines edited in AvrXa7 (a) or TalC (b) EBEs. On top, the 118 bp‐*SWEET14* sequence corresponds to the −328 to −211 promoter fragment relative to the ATG start codon. These mutant alleles were brought to the homozygous stage. Allele names are provided on the left side of each sequence and the nature of the associated mutation on the right. Dashed lines below the *SWEET14* wild‐type sequence represent the binding sites of the TALEN pairs used in this study. Deletions and insertions are represented by black dashes and blue letters, respectively.

To modify three EBEs within the *SWEET14* promoter (the two overlapping AvrXa7 and Tal5 EBEs and the more upstream TalC EBE), we conducted two sets of experiments, targeting AvrXa7 and TalC EBEs, respectively. To do so, we assembled two TALEN pairs designed to recognize sequences on both sides of each EBE (Figure 1, black dashed lines). For each pair, the left (L‐) and right (R‐) TALEN‐encoding genes were cloned into distinct binary vectors and subsequently introduced into *Agrobacterium tumefaciens*. The two resulting *A. tumefaciens* strains were mixed prior to transformation of the Kitaake rice cultivar. PCR‐based analysis of T0‐regenerated seedlings showed that 84% and 90% of the plants studied (for AvrXa7 and TalC EBE mutagenesis, respectively) had integrated both transgenes. To characterize edition events, 342 base‐pair (bp) PCR fragments amplified from double transgenic individuals and encompassing the three EBEs were Sanger sequenced and the resulting chromatograms were manually deconvoluted to resolve the sequence of the edited alleles. We found that 51% and 30% of the plants carrying both T‐DNAs (for AvrXa7 and TalC EBEs, respectively) were edited at one or both gene copies. Mutations were either deletions of up to 51 bp (68% and 73% of all mutations for AvrXa7 and TalC EBEs, respectively), insertions of up to 22 bp (7% in both cases) or combined deletion plus insertion events (26% and 19%). Altogether, we obtained 41 distinct mutations within AvrXa7 EBE, 13 of which also affected the Tal5 EBE, and 26 distinct mutations within TalC EBE (Table 1). We selected several T0 lines, corresponding to overall 19 AvrXa7‐Tal5 EBE alleles (Figure 1a) and 16 TalC EBE alleles (Figure 1b), to study mutation transmission and segregation at the T1 generation (Table 1, see also Supplementary Tables S1 and S2). *SWEET14* promoter sequencing of the T1 plants revealed that all mutations were transmitted to the next generation and that their segregation pattern was consistent with classical Mendelian inheritance, with a few exceptions. Three lines carrying biallelic mutations in T0 produced T1 progenies with one to two additional mutations, suggesting that TALENs were still active on the edited sequences. Transgenic T1 plants carrying each mutation at the homozygous state were selected and propagated. At that stage, we also selected individuals deprived of both T‐DNAs using a PCR approach, which was successful for 10 of 11 AvrXa7 EBE‐edited T1 lines, and five of 10 TalC EBE‐edited T1 lines (Table 1, S1 and S2). All phenotypic analyses described below were performed on T2 or T3 plants that carried homozygous mutations, as confirmed by promoter sequencing.

**Table 1:** Summary of the results obtained for editing of AvrXa7 and TalC EBEs in *O. sativa* cv. Kitaake using TALENs

	AvrXa7 EBE	TalC EBE
Number of T0 plants studied	135	171
Number of T0 plants carrying both transgenes	113	154
Number of T0 edited plants retrieved	58	46
Edition efficiency (edited *vs*. carrying both transgenes)	51%	30%
Number of independent T0 lines retrieved	30	23
Number of distinct edited alleles and classification (deletions/insertions/combined events)	41 (27/3/11)	26 (19/2/5)
Range of the deletions	−1 to −51 bp	−3 to −24 bp
Range of the insertions	+1 to +23 bp	+1 to +10 bp
Number of independent T0 lines genotyped at the T1 generation	11	10
Number of independent edited alleles available at the homozygous stage (deletions/insertions/combined events)	19 (15/1/3)^a^	16 (13/2/1)^b^
Number of transgene‐free T1 lines carrying a unique edited allele at the homozygous stage	10	5

a Alleles represented in Figure 1a (see also detailed information in Table S1).

b Alleles represented in Figure 1b (see also detailed information in Table S2).

### Mutations within AvrXa7 and Tal5 EBEs lead to resistance against bacterial strains relying on the corresponding effectors

**Figure 2:**
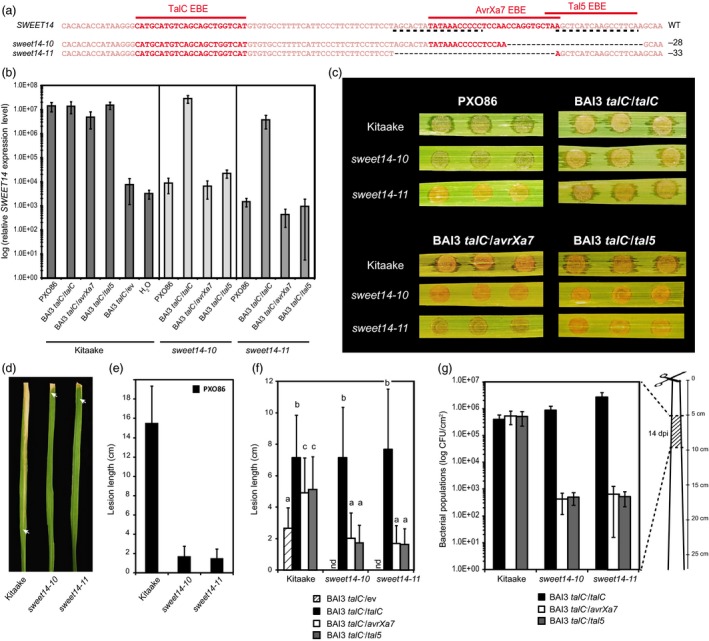
Functional analysis of two Kitaake edited lines carrying deletions in AvrXa7 and/or Tal5 EBEs. (a) Location of the studied mutations on the *SWEET14* promoter (same fragment as in Figure 1). (b) *SWEET14* expression pattern obtained by RT‐qPCR 2 days post‐leaf infiltration with the indicated strains. The graph uses a base‐10 logarithmic scale. Bars represent the average expression obtained from four independent RNA samples, with standard deviation. (c) Water‐soaking symptoms obtained after leaf infiltration with the indicated strain. Pictures were taken 5 days post‐inoculation (dpi). (d and e) Qualitative (d) and quantitative (e) evaluation of the disease symptoms obtained 14 days post‐leaf clipping inoculation with the PXO86 wild‐type strain. For d, lesions were photographed at 14 dpi and arrow heads indicate the end of the lesion. On e, bars represent the average and standard deviation obtained from N > 30 symptomatic leaves. (f) Lesion length measured 21 days post‐leaf clipping inoculation with the indicated BAI3 derivative strains. Bars represent the average and standard deviation obtained from N > 20 symptomatic leaves. The letters above the bars represent the result of a Tukey's HSD statistical test. Identical letters indicate means that are not significantly different from each other (α = 0.05). ‘nd’, not determined. (g) Bacterial populations extracted at 14 days post‐leaf clipping inoculation from the 5‐cm leaf segment as depicted on the cartoon. The graph uses a base‐10 logarithmic scale. Bars represent the average and standard error obtained from four independent leaf samples.

It was previously shown that TALEN‐mediated mutations within AvrXa7 EBE are able to defeat an *Xoo pthXo1* mutant strain expressing *avrXa7* in *trans* (Li *et al*., 2012). Furthermore, our group recently demonstrated that in some accessions of the African wild rice *O. barthii* and its domesticated progeny (*Oryza glaberrima*), *xa41*, a naturally occurring *SWEET14* allele consisting in a deletion encompassing AvrXa7 and Tal5 EBEs, confers resistance to various *Xoo* strains including an *avrXa7* expressing strain (Hutin *et al*., 2015b). To check whether (i) engineered mutations within AvrXa7 EBE could also confer resistance to the original PXO86 wild‐type strain from which *avrXa7* was isolated, and (ii) mutations resembling *xa41* could also confer resistance to *avrXa7* or *tal5* expressing strains in an *Oryza sativa* background, we focused on two Kitaake edited lines, carrying the *sweet14‐10* (a 28‐bp deletion equivalent to the 18‐bp *xa41* deletion) and *sweet14‐11* (a 33‐bp deletion removing the entire AvrXa7 EBE and the first two base pairs of Tal5 EBE) alleles, respectively (Figure 2a).

*SWEET14* expression was induced more than 1500‐fold upon infiltration with PXO86 or a BAI3 *talC* ^−^ insertion mutant carrying a vector encoding *talC*,* avrXa7* or *tal5*. Whereas this induction was not affected in either of the two edited lines in response to BAI3, it was abolished in both of them after infection with PXO86, BAI3 *talC* ^−^/*avrXa7* and BAI3 *talC* ^−^/*tal5* (Figure 2b). This suggests that both *sweet14‐10* and *sweet14‐11* mutations prevent EBE recognition by the AvrXa7 and Tal5 TALEs, but retain TalC responsiveness. Interestingly, these data further show that a mutation of the first two base pairs of Tal5 EBE is sufficient to prevent recognition by this effector.

Disease caused by *Xoo* bacteria is typically assessed using two methods: the first one consists of syringe infiltration of bacteria into leaves and following the development of water‐soaked lesions around the infiltration area. The second approach, called leaf clipping, consists of cutting the tip of the leaves using scissors previously dipped in the bacterial suspension, and measuring the length of the chlorotic to necrotic lesions developing along the leaf blade. Using these methods, both edited lines were unable to develop water‐soaking or vascular lesions in response to PXO86 bacteria (Figure 2c, d and e). When testing the BAI3 *talC* ^−^ complemented strains, only the strain expressing TalC was virulent on the edited lines (Figure 2c, f and g), suggesting that both *sweet14‐10* and *sweet14‐11* alleles are able to defeat strains relying on AvrXa7 and Tal5.

As previously observed on the rice accession IR24, expression of neither *talC*,* avrXa7* nor *tal5* from a plasmid is able to fully complement a BAI3 *talC* ^*−*^ mutant (Streubel *et al*., 2013). At 21 dpi, a time point where disease was more pronounced, infection with the strain delivering TalC triggered similar symptoms in Kitaake wild‐type and edited plants. However, lesions caused by *avrXa7* or *tal5*‐complemented strains were >twofold shorter in the edited lines *vs*. wild‐type plants (Figure 2f). At 14 dpi, quantification of bacterial populations in a 5‐cm leaf section located 5 cm away from the clipping site showed that both edited lines supported ca. 1000‐fold less bacterial colonization than wild‐type plants when using *avrXa7* or *tal5*‐complemented strains (Figure 2g). These data, relying on a distinct set of bacterial strains, distinct plant genetic backgrounds and novel edited alleles confirm and broaden previous studies showing that loss of *SWEET14* induction in AvrXa7 or Tal5 EBE‐edited lines correlates with loss of susceptibility in response to the TALEs AvrXa7 and Tal5 (Hutin *et al*., 2015b; Li *et al*., 2012).

### *SWEET* alleles mutated in the TalC EBE lose TalC responsiveness but do not confer resistance

**Figure 3:**
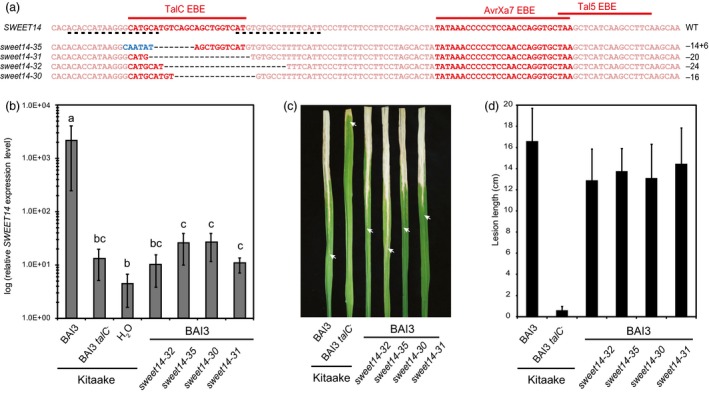
Functional analysis of four Kitaake edited lines carrying mutations in TalC EBE. (a) Location of the studied mutations on the *SWEET14* promoter (same fragment as in Figure 1). (b) *SWEET14* expression pattern obtained by RT‐qPCR 2 days post‐infiltration with the indicated strains. Bars represent the average expression obtained from four independent biological experiments, each including four independent RNA samples (16 in total), with standard deviation. The letters above the bars represent the result of a Tukey's HSD statistical test. Identical letters indicate means that are not significantly different from each other (α = 0.05). (c and d) Lesion length photographed (c) or measured (d) 14 days post‐leaf clipping inoculation with the BAI3 wild‐type or *talC* mutant strain. On (c), arrow heads indicate the end of the lesion. On (d), bars represent the average and standard deviation obtained from N > 30 symptomatic leaves.

We next studied four lines carrying independent mutations within TalC EBE (Figure 3a). *SWEET14* expression in the four edited lines in response to BAI3 was reduced about 1000‐fold relative to Kitaake and resembled that of wild‐type plants inoculated with the BAI3 *talC* ^*−*^ strain (Figure 3b), showing that TALEN‐mediated EBE disruption successfully rendered this gene non‐responsive to TalC. Surprisingly, symptoms caused by wild‐type BAI3 bacteria on the TalC EBE‐edited lines were hardly reduced compared to those observed on Kitaake plants, irrespective of the inoculation method used. Vascular lesions measured 14 days after leaf clipping were marginally shorter in two independent replicates of the experiment shown in Figure 3c and d or basically identical in one replicate of the same experiment (not shown). Furthermore, water‐soaking symptoms at 6 dpi were either unaltered or only slightly reduced (Supplementary Figure 1). These results show that loss of *SWEET14* induction upon TalC delivery in Kitaake plants fails to confer disease resistance.

**Figure 4:**
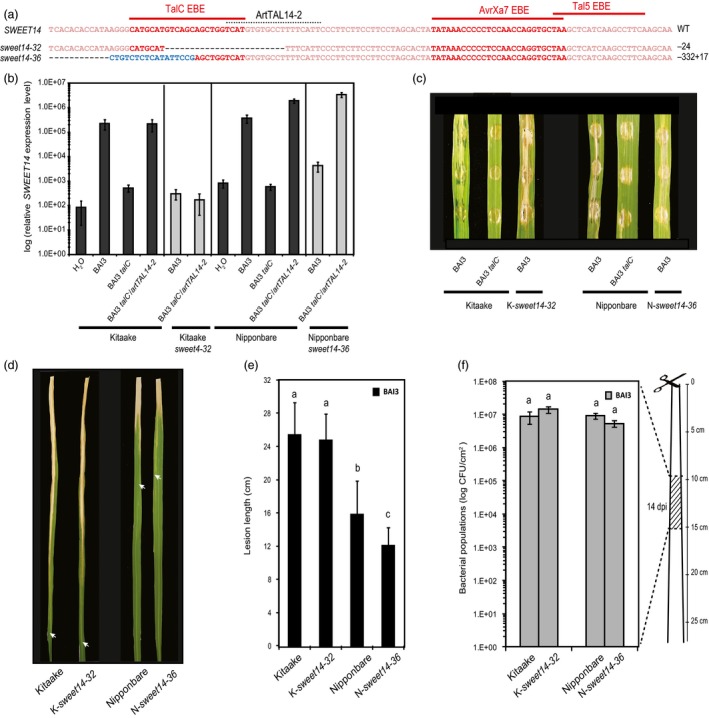
Functional analysis of edited rice lines carrying mutations in TalC EBE in the Kitaake (*sweet14‐32*) or Nipponbare (*sweet14‐32*) backgrounds. (a) Location of the studied mutations on the *SWEET14* promoter (same fragment as in Figure 1). (b) *SWEET14* expression pattern obtained by RT‐qPCR 2 days post‐infiltration with the indicated strains. The graph uses a base‐10 logarithmic scale. Bars represent the average expression obtained from four independent RNA samples, with standard deviation. (c) Symptoms obtained 5 days post‐leaf infiltration of the indicated strain. The letter in front of each allele indicates the cultivar, K‐ for Kitaake and N‐ for Nipponbare. (d and e) Lesion length photographed (d) or measured (e) 14 days post‐leaf clipping inoculation of the BAI3 wild‐type strain. On (d), arrow heads indicate the end of the lesion. On (e) bars represent the average and standard deviation obtained from N > 30 symptomatic leaves, and letters above the bars represent the result of a Tukey's HSD statistical test. Identical letters indicate means that are not significantly different (α = 0.05). (f) Bacterial populations extracted at 14 days post‐leaf clipping inoculation from a 5‐cm leaf segment as depicted on the cartoon. The graph uses a base‐10 logarithmic scale. Each bar represents the average obtained from four independent samples, with standard error. A Tukey's HSD test performed on these data indicated no statistically significant differences (α = 0.05).

As the genome sequence of cultivar Kitaake is not available, we cannot exclude that a gene duplication of *SWEET14* had happened in this genetic background. We therefore examined whether the ‘retained susceptibility’ phenotype was restricted to edited plants of this cultivar or shared by other accessions, thus representing an intrinsic feature of TalC‐triggered susceptibility. To this end, we used the same TALEN pair as before to mutagenize TalC EBE in the reference *O. sativa* Nipponbare background, and studied a line carrying an homozygous −332 + 17 indel affecting the 5′ end of TalC EBE (Figure 4a), named *sweet14‐36*. In these analyses, we used the Kitaake/*sweet14‐32* line for comparison. Gene expression studies performed in the same conditions as before confirmed non‐induction of *SWEET14* in response to BAI3 in the edited Nipponbare line, *sweet14‐36*, compared to wild‐type plants (Figure 4b). In leaf clipping assays, even if lesions obtained on Nipponbare were shorter than those typically measured on Kitaake in response to BAI3, TalC EBE disruption in Nipponbare *sweet14‐36* only allowed a very slight gain of resistance in terms of symptom development (Figure 4d and e). Bacterial growth assays performed with all lines at 14 dpi did not reveal any significant difference in bacterial population sizes in both edited lines *vs*. wild‐type plants in the 10‐ to 15‐cm leaf section beyond the inoculation site (Figure 4f). These data demonstrate that Nipponbare behaves similar to Kitaake and that a hypothesized *SWEET14* gene duplication cannot account for the uncoupling between disease resistance and loss of TalC responsiveness at the *SWEET14* locus. We therefore conclude that loss of *SWEET14* induction alone is not sufficient to confer strong resistance to *talC*‐expressing bacteria.

### The susceptibility of TalC EBE‐edited lines does not require *SWEET14* induction but specifically depends on *talC*

The unexpected susceptibility of TalC EBE‐edited lines could be an unintended consequence of the editing process due to ‘off‐target’ editing or tissue culture somaclonal variation. For example, the immune system of an edited rice line may have been accidentally modified, leading to strong susceptibility to *Xoo* infection. Alternatively, the regulation of genetically redundant susceptibility gene(s) whose activity can compensate for the loss of *SWEET14* induction may have been affected. If susceptibility to BAI3 resulted from the misregulation of such susceptibility gene(s), we would expect complementation of the loss of TalC activity in a BAI3 *talC* ^*−*^ mutant, rescuing virulence on the *sweet14‐32* line.

**Figure 5:**
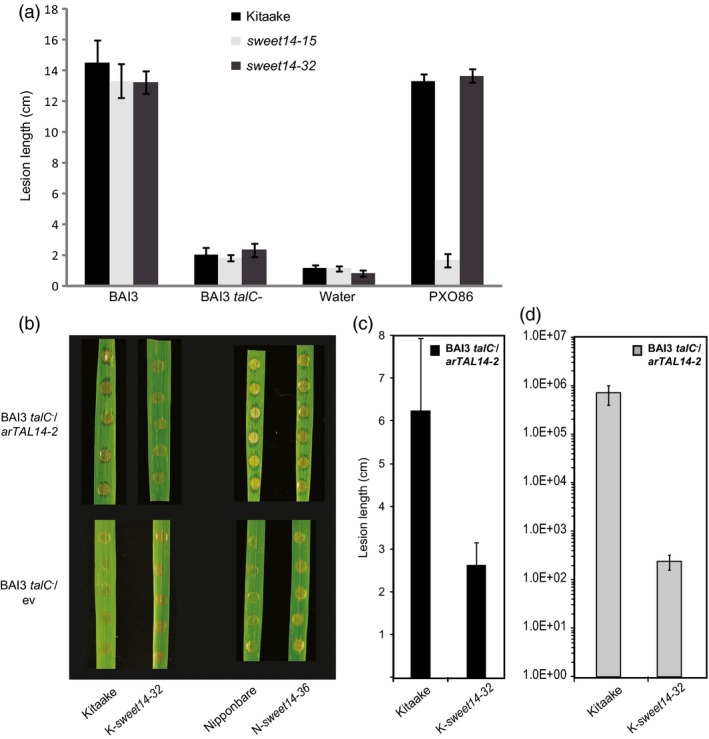
TalC EBE‐edited plants are resistant to the BAI3 *talC* mutant and to bacteria expressing ArtTAL14‐2. (a) Lesion length measured 14 days post‐leaf clipping inoculation of the edited lines *sweet14‐15* and *sweet14‐32* together with the control background genotype Kitaake. Bacterial or mock treatments are indicated under the *x*‐axis. Bars represent the average and standard deviation obtained from N > 3 symptomatic leaves. This experiment was repeated three times with similar results. (b) Water‐soaking symptoms obtained after infiltration of the BAI3 *talC* mutant expressing in *trans* the artificial TAL gene *artTAL14‐2* (binding site represented in Figure 4a) or the corresponding empty vector (ev). Pictures were taken 5 dpi. (c) Lesion length measured 21 days post‐leaf clipping inoculation of the Kitaake edited line with BAI3 *talC* ^−^/*artTAL14‐2* strain. Bars represent the average and standard deviation obtained from N > 20 symptomatic leaves. (d) Bacterial populations extracted at 14 days post‐leaf clipping inoculation from a 5‐cm leaf segment collected as depicted on the cartoon in Figure 2g. The graph uses a base‐10 logarithmic scale. Each bar represents the average obtained from four independent samples, with standard error.

To address this question, we first used leaf clipping to inoculate the *sweet14‐32* line, the AvrXa7/Tal5 EBEs‐edited line *sweet14‐15* (Figure 1a) and wild‐type Kitaake. In addition to the BAI3 *talC* ^*−*^ mutant, these plants were also challenged with BAI3 and PXO86 as positive controls and water as a negative control. As shown in Figure 5a, wild‐type BAI3 caused similarly severe BLB symptoms with lesion lengths reaching more that 12 cm on all three host genotypes while leaves inoculated with the BAI3 *talC* ^*−*^ mutant remained essentially symptomless, comparable to the water controls. Lesions produced by PXO86 on *sweet14‐32* and Kitaake leaves were similar to those caused by BAI3. As observed before on AvrXa7/Tal5 EBE‐edited lines (Figure 2), PXO86 did not cause lesions on *sweet14‐15* plants. These results demonstrate that susceptibility of the edited lines to BAI3 depends on TalC and that the TalC EBE‐edited line *sweet14‐32* is not generally impaired in immunity to *Xoo* infection.

To further examine whether these lines can mount resistance to an *Xoo* infection and to uncouple *SWEET14* induction from other TalC activities, we used a *talC* ^−^ mutant strain expressing an artificial *SWEET14*‐targeting TALE, *artTAL14‐2* (Streubel *et al*., 2013). Because of the position of its binding site, which is essentially unrelated to and located downstream of the TalC EBE (see the dotted line in Figure 4a), *SWEET14* promoter recognition by ArtTAL14‐2 in the Kitaake/*sweet14‐32* line should also be abolished and prevent gene induction in response to this strain. RT‐qPCR experiments confirmed that *SWEET14* expression in the edited Kitaake line was not induced upon infection with BAI3 or BAI3 *talC* ^−^/*artTAL14‐2* (Figure 4b). By contrast, strong induction was obtained in the edited Nipponbare line *sweet14‐36* carrying an intact ArtTAL14‐2 EBE in the promoter of *SWEET14* (Figure 4b). In parallel, we assessed the resistance levels of the TalC EBE‐edited lines in response to BAI3 *talC* ^−^/*artTAL14‐2*. While water‐soaking symptoms elicited by this strain after infiltration of the Nipponbare/*sweet14‐36* line were similar to the corresponding wild‐type plants, they were dramatically reduced on Kitaake/*sweet14‐32* plants (Figure 5b). Following leaf clipping, lesion length was reduced by half (Figure 5c) and bacterial population sizes were reduced more than 1000‐fold (Figure 5d). Hence, although the *artTAL14‐2* construct only partially complemented the BAI3 *talC* ^−^ strain in leaf clipping assays as previously reported (Streubel *et al*., 2013), Kitaake/*sweet14‐32* plants were significantly and reproducibly more resistant to BAI3 *talC* ^−^/*artTAL14‐2* compared to wild‐type plants.

Altogether, these data show that non‐induction of *SWEET14* upon delivery of ArtTAL14‐2 in the *sweet14‐32* line correlates with elevated disease resistance levels. This finding contrasts with results obtained with *talC* where *SWEET14* induction and susceptibility are clearly uncoupled (Figure 4). In conclusion, the *sweet14‐32* line is not generally impaired in immunity to *Xoo* infection and TalC‐dependent disease occurs independent of *SWEET14* gene induction.

### The susceptibility of TalC EBE‐edited lines does not appear to be due to induction of a clade‐III *SWEET* susceptibility gene

The *talC*‐dependent susceptibility of TalC EBE‐edited lines prompted us to consider the possibility that the TalC regulon extends to other, genetically redundant gene(s) beyond *SWEET14. SWEET genes* form a multigenic family in rice. Notably, clade‐III *SWEETs* have been shown to be functionally equivalent susceptibility genes in the rice–*Xoo* interaction (Streubel *et al*., 2013). Clade‐III *SWEETs* other than *SWEET14* could thus potentially act as redundant, TalC‐targeted susceptibility genes.

**Figure 6:**
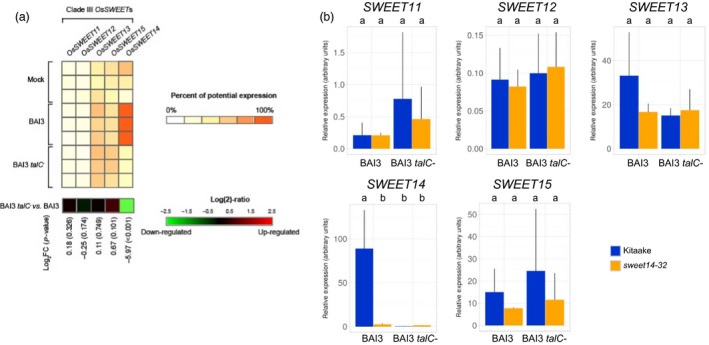
Expression of the five clade‐III *OsSWEET* genes in response to BAI3 or a *talC* mutant. (a) Gene expression derives from previously published microarray data and analysed using Genevestigator. The heat‐map displays the percent of potential expression for *SWEET11* (*LOC_Os08 g42350*), *SWEET12* (*LOC_Os03 g22590*), *SWEET13* (*LOC_Os12 g29220*), *SWEET15* (*LOC_Os02 g30910*) and *SWEET14* (*LOC_Os11 g31190*). Each row refers to an independent replicate of the microarray experiment. The last row of the heat‐map represents the log_2_ of the expression ratio between conditions ‘BAI3 *talC* ^−^ mutant’ *vs*. ‘BAI3 WT’, with associated *P*‐value (into brackets). (b) Clade‐III *SWEET* genes expression was measured by RT‐qPCR 2 days post‐infiltration of the *sweet14‐32* line or the Kitaake background with the wild‐type BAI3 or the BAI3 *talC* ^−^ mutant strains. Bars represent average expression obtained from three independent RNA samples, with standard deviation. Letters above the bars represent the result of a Tukey's HSD test. Identical letters correspond to means that are not significantly different from each other (α = 0.05). This experiment was repeated three times with similar results.

To test this, we first examined available transcriptomic data obtained after infection with BAI3 or a BAI3 *talC* ^−^ mutant (Yu *et al*., 2011). We found that among the five clade‐III *SWEETs* (*SWEET11* to *SWEET15*), only *SWEET14* is significantly and strongly induced in a *talC*‐dependent manner upon BAI3 infection (Figure 6a). This observation indicates that TalC does not target any additional clade‐III *SWEET* gene beyond *SWEET14* in the susceptible Nipponbare cultivar. Even if unlikely, we cannot *a priori* exclude the possibility that TalC binds to an alternate, low‐affinity DNA box at another genomic locus when its primary target EBE is destroyed, thus directly or indirectly inducing another clade‐III *SWEET* gene and compensating the loss of *SWEET14* induction. In order to examine this possibility, we performed RT‐qPCR to compare clade‐III *SWEET* gene expression following leaf infiltration of the TalC EBE‐edited line *sweet14‐32* versus wild‐type Kitaake. First, to ensure that the qPCR primer pairs designed to monitor *SWEET* gene expression (Streubel *et al*., 2013) also perform well in our genetic backgrounds, we confirmed the induction of individual clade‐III *SWEET* genes upon infiltration with a *Xoo* strain expressing a TALE (natural or artificial) targeting the corresponding *SWEET* gene (Supplementary Figure 2). Next, we infiltrated the *sweet14‐32* line and Kitaake with either BAI3 or the BAI3 *talC* ^−^ mutant and monitored expression of *SWEET11* to *SWEET15* (Figure 6b). As observed previously (Figure 4), *SWEET14* was highly induced in Kitaake 48 h post‐BAI3 inoculation as compared to the BAI3 *talC* ^−^ strain, and this induction was abrogated in the *sweet14‐32* edited background. For the remaining four clade‐III *SWEET* genes, testing for mean differences across bacterial strain–rice genotype combinations did not reveal any statistically significant differences in transcript abundance (Figure 6b) in any of the three biological experiments that were independently performed. Thus, retained susceptibility of edited lines at the TalC EBE of *SWEET14* is unlikely to result from TalC‐mediated induction of a clade‐III *SWEET* gene.

## Discussion

Given enormous potential, genome editing has attracted biologists of a broad range of disciplines and underwent an explosive development in the past years. In plants, while genome editing strategies including zinc‐finger nucleases, TALENs or CRISPR‐Cas systems have been deployed in a number of studies, most of them have essentially addressed method implementation and analyses of the nature and heritability of the generated mutations. Yet, very few studies have provided significant added value to functional genomics and/or breeding.

Using TALENs, we have efficiently edited the promoter of a central BLB susceptibility gene at two distinct target sequences. In a simple experimental set‐up based on two TALEN‐expressing transgenes and two *A. tumefaciens* strains, we have obtained mutation frequencies (up to 51%) that had not been reached in studies based on expression of both TALENs from a single T‐DNA: 19‐36% (Zhang *et al*., 2013), 21%–25% (Zhang *et al*., 2016) or 30% (Shan *et al*., 2015).

The nature of the mutations obtained in our study, as well as the proportion of deletions *vs*. insertions or indels, followed a distribution resembling previous reports (Zhang *et al*., 2016), although it is interesting to note that we did not obtain any event of the ‘substitution’ type. Instead, striking differences are observed regarding mutation zygosity. In previous studies, the presence, or even prevalence, of chimeras (at least three different sequences including the wild‐type sequence) in T0‐regenerated plantlets, suggests that TALEN‐induced mutations likely occur rather late during differentiation of the embryogenic cells into plants (Shan *et al*., 2015; Zhang *et al*., 2016). In contrast, all our T0 edited plants carried monoallelic or biallelic mutations, suggesting that the TALEN pairs used have worked efficiently at an early stage of the transformation process. Driving TALENs transcription with the strong maize ubiquitin promoter may have contributed to the absence of chimera and high level of biallelic edition in the regenerated plants in our experiments.

Transmission to subsequent generations through Mendelian inheritance was achieved in all published studies including ours. Like previous reports (Shan *et al*., 2015), additional mutations were in a few cases detected in the offspring of heterozygous T0 lines carrying biallelic mutations, suggesting that in some cases, in particular when TALEN‐binding sites were not disrupted in the first round of mutations, further TALEN DBS activity has occurred in gamete progenitor cells leading to T1 seeds. Furthermore, our simple but highly efficient ‘two‐strain’ strategy does not preclude the possibility to use T‐DNA segregation at the T1 generation to identify transgene‐free edited plants. This was achieved for a large proportion of our edited lines (Supplementary Tables 1 and 2).

TALEN‐induced mutations engineered at AvrXa7 EBE in the Kitaake background were shown to render rice plants resistant to a *pthXo1* mutant expressing *avrXa7* or *pthXo3* (Li *et al*., 2012). In addition, a 18‐bp deletion encompassing AvrXa7 and Tal5 EBEs in *O. glaberrima* and some accessions of *O. barthii* confers resistance to *avrXa7‐* or *tal5*‐expressing bacteria (Hutin *et al*., 2015b). Using systematic EBE editing on *O. sativa* cv. Kitaake plants, we show that indels affecting AvrXa7 and Tal5 EBEs can defeat the corresponding strains by making the major susceptibility gene *SWEET14* unresponsive to the TALEs. Collectively, three studies therefore provide evidence that AvrXa7 and Tal5 are major virulence TALEs, with *SWEET14* being their unique susceptibility target. In addition, our work offers a large repertoire of alleles that can be used in breeding for resistance to AvrXa7‐ or Tal5‐expressing strains. Because the function of dominant *S* genes is critical for bacterial virulence, mutated *S* gene alleles have been proposed as a potentially more durable resistance strategy compared to dominant resistance executor (*E*) genes (Gawehns *et al*., 2013; Gust *et al*., 2010; Iyer‐Pascuzzi and McCouch, 2007; Leach *et al*., 2001). Nonetheless, TALE‐dependent loss of susceptibility can be overridden through multiple evolutionary routes (Hutin *et al*., 2015a). Predicting the durability of natural or engineered recessive resistance and long‐term field testing of resistant lines remains a great challenge for EBE‐based resistance breeding.

Contrary to AvrXa7‐ or Tal5 EBEs, the biological significance of TalC‐triggered *SWEET14* induction has never been addressed so far. Here, we provide a set of *SWEET14* alleles disrupted in TalC EBE and their functional characterization. Unexpectedly, editing of this EBE had only a little impact, if any, on resistance, as opposed to modification of AvrXa7 or Tal5 EBEs, which results in a strong gain in resistance. While *SWEET14* induction by TalC or artificial TALEs is sufficient to trigger disease (Streubel *et al*., 2013; Yu *et al*., 2011), preventing TalC‐mediated *SWEET14* activation does not compromise plant susceptibility. Similar results were obtained in Kitaake and in the reference rice cultivar Nipponbare which carries a single *SWEET14* gene, thereby ruling out the possibility that a gene duplication event in Kitaake has masked the effect of the EBE disruption.

The unaffected susceptibility of a TalC EBE‐edited line in response to BAI3 could originate from unspecific modifications of the genome or epigenome introduced either by the nucleases (off‐targets) or by the genetic transformation process itself (somaclonal variation), that could translate into impaired antibacterial immunity or compensation for the absence of *SWEET14* induction (through enhanced apoplastic relase of carbohydrates for example). However, the TalC EBE‐edited line *sweet14‐32* was as resistant as Kitaake against a BAI3 *talC* ^−^ mutant strain (Figure 5a) and even more resistant than the wild type when this strain carried *artTAL14‐2* (Figure 5b‐d). These findings clearly argue against unintended modifications of the genome elsewhere than at the *SWEET14* promoter being causal to the retained susceptibility phenotype of TalC EBE‐edited lines.

Importantly, both TalC and ArtTAL14‐32 fail to mediate *SWEET14* induction in the *sweet14‐32* line because their cognate EBEs are largely deleted in this allele. Yet, while susceptible to wild‐type BAI3 bacteria expressing TalC (Figure 3c‐d), this edited line was resistant to BAI3 *talC* ^*−*^ bacteria expressing the artificial TALE (Figure 5b‐d), demonstrating that ArtTAL14‐2 and TalC have separable virulence activities. Because both proteins possess distinct RVD arrays, and are, therefore, unlikely to share the same targets beyond *SWEET14*, we favour the hypothesis that TalC targets additional, genetically redundant, susceptibility gene(s). Genetic interplay between TalC susceptibility targets may involve some degree of additive effect rather than strict redundancy because the BAI3 *talC* ^−^/*talC* strain reproducibly induced stronger symptoms and its population reached higher levels *in planta* compared to BAI3 *talC* ^−^ strains expressing *avrXa7* or *tal5* (as exemplified in Figure 2f on Kitaake). Members of the clade‐III *SWEET* family were obvious candidates to be tested as susceptibility genes in the absence of *SWEET14* induction. However, neither analysis of public microarray data obtained in a wild‐type Nipponbare background (Figure 6a) nor our own transcript profiling in the Kitaake TalC EBE‐edited background (Figure 6b) detected significant *talC*‐dependent upregulation of a clade‐III *SWEET* besides *SWEET14*. Yet, even if very unlikely, the possibility that a clade‐III *SWEET* gene is a *bona fide* TalC target cannot be completely excluded on the sole basis of this data. Clearly, more work is required to unambiguously identify the redundant TalC target(s) and to validate its/their function as susceptibility gene(s).

Although major virulence TALEs are known to have direct targets with no or unexplored biological activity in addition to their primary *S* gene target (Boch *et al*., 2009; Cernadas *et al*., 2014; Li *et al*., 2012), no TALEs have been reported so far to possess more than a single biologically significant target. It will be critical to determine whether this feature is shared by other TALEs from *Xanthomonas* strains infecting crops. If so, resistance strategies based on engineering single TALE‐unresponsive *S* gene alleles may not be as straightforward as originally anticipated (Hutin *et al*., 2015a; Li *et al*., 2012) and will require a much finer understanding of virulence TALE targets.

## Experimental procedures

### Design and assembly of TALENs targeting AvrXa7 and TalC EBEs

We designed TALENs pairs with DNA‐binding domains composed of 16–18 repeats. Our main criteria for selecting both binding sequences were the following: (i) a T at position zero, (ii) a 15‐bp spacer region to allow *Fok*I dimerization and (iii) a minimum of three strong RVDs (HD or NN) in the repeat array (corresponding in the target sequence to C and G, respectively; Streubel *et al*., 2012). Modular assembly in a compatible ENTRY vector was performed using the GoldenTal method (Geiβler *et al*., 2011). The N‐terminal domain of the TALENs contained a portion of TALE Hax3 (amino acids 153–288), a SV40 nuclear localization signal and an epitope tag (left TALEN: c‐myc; right TALEN: HA). The C‐terminal domain contained Hax3 amino acids 1–63 and an heterodimeric ‘sharkey’ *Fok*I nuclease domain [left TALEN: DS variant; right TALEN: RR variant (Guo *et al*., 2010)]. The left and right TALENs were inserted between the maize ubiquitin promoter and the NOS terminator into the pCAMBIA2300 (geneticin resistance) and pCAMBIA5300 (hygromycin resistance) binary plasmids (http://www.cambia.org/), respectively, using GATEWAY^®^ cloning (INVITROGEN). The resulting constructs were mobilized into *Agrobacterium tumefaciens* strain EHA105 by electroporation.

### Rice stable transformation with TALEN constructs

TALEN expression in rice was accomplished through *A. tumefaciens*‐mediated stable transformation, as previously described (Sallaud *et al*., 2003). Two *A. tumefaciens* strains, each harbouring one of the two TALENs, were mixed prior to transformation of *Oryza sativa* L. ssp. *japonica* (cvs. Kitaake or Nipponbare) *via* co‐culture of bacteria with seed‐embryo calli. The calli were transferred to plates containing 50 mg/L hygromycin for 2–3 weeks, until hygromycin‐resistant cell lines develop, then for three additional weeks on plates supplemented with both antibiotics (50 mg/L hygromycin and 100 mg/L geneticin), in order to select transformed cell lines carrying both T‐DNAs.

### Molecular analysis of T0 plants and chromatogram‐based detection of mutations

Leaf samples were collected from T0 plants and subjected to DNA extraction using a standard MATAB‐based protocol (Romero *et al*., 2014). A portion of the *OsSWEET14* promoter (342 bp in wild‐type plants) was amplified by PCR using primers 5′‐TCCAGGGTCACACACCATAAG and 5′‐TGCAGCAAGATCTTGATTAACTA. For analysis of the Nipponbare allele *sweet14‐36*, the reverse primer used was 5′‐TTGCGGCTCATCAGTTTCTC). After DNA sequencing of the PCR products, chromatograms that harboured off‐set traces due to sequence heterozygosity were resolved manually and sequences of the edited alleles were deduced by alignment to the wild‐type promoter sequence. The presence of the T‐DNA originating from pCAMBIA5300 was monitored by PCR using the 5′‐CTGAACTCACCGCGACGTCTG and 5′‐GGCGTCGGTTTCCACTATCG primers specific for the Hpt hygromycin resistance marker gene, and presence of the T‐DNA originating from pCAMBIA2300 using the 5′‐GCGATAGAAGGCGATGCG and 5′‐CCGGCTACCTGCCCATTCGA primers specific for the NptII geneticin resistance marker gene.

### Bacterial strains, plant inoculations and growth of bacteria *in planta*

All *Xoo* strains used in this study are published: wild‐type BAI3 (Gonzalez *et al*., 2007), wild‐type PXO86 (Vera Cruz, 1989), PXO99A (Hopkins *et al*., 1992), BAI3 *talC* ^−^ mutant (Yu *et al*., 2011) and BAI3 *talC* ^−^ complemented with plasmids containing *talC*,* avrXa7*,* tal5*,* artTAL12‐2*,* artTAL13‐2*,* artTAL14‐2* or *artTAL15‐1* coding sequences (Streubel *et al*., 2013). Rice cultivation and disease assays were performed as previously described (Hutin *et al*., 2015b; Yu *et al*., 2011). Bacteria were inoculated at an optical density (OD_600_) of 0.5 (infiltrations) or 0.4 (leaf clipping) in water. For quantification of bacterial populations, 5‐cm leaf sections were collected at 5 dpi. Each processed sample contained leaf sections from three independent inoculations, and four to six independent samples were processed as per condition. Samples were frozen in liquid nitrogen, ground using metal beads, diluted in water and spotted on PSA medium containing the appropriate antibiotics.

### Gene expression analyses

A 4‐cm leaf section was entirely infiltrated, and collected at 48 h post‐inoculation (hpi) for RNA extraction. Each individual sample contained three independent infiltration areas, and three to four independent replicate samples were processed. After sample grinding, total RNA was extracted from plant leaves using TRI‐reagent (EUROMEDEX), and further purified using the RNA Clean‐Up & Concentration kit (ZYMO RESEARCH). After TURBO DNase treatment (AMBION), 3 μg RNA were reverse transcribed into cDNA using SuperScriptIII (INVITROGEN). All gene expression studies were performed by quantitiative real‐time PCR in a LightCycler (ROCHE), using SYBR‐based Mesa Blue qPCR Mastermix (EUROGENTEC). Average transcript levels were calculated using the ∆Ct method from three to four independent cDNA samples using the *OsEF‐1*α gene for normalization. *OsSWEET14* transcript was studied using primers 5′‐ACTTGCAAGCAAGAACAGTAGT and 5′‐ATGTTGCCTAGGAGACCAAAGG and *OsEF‐1*α transcript using primers 5′‐GAAGTCTCATCCTACCTGAAGAAG and 5′‐GTCAAGAGCCTCAAGCAAGG.

## Conflict of interest

The authors declare no conflict of interest.

## Supporting information


**Figure 1** Water‐soaking symptoms obtained for the four TalC EBE‐edited lines studied at five days after infiltration with BAI3 wild‐type bacteria.
**Figure 2** Clade‐III SWEET genes upregulation in response to bacterial strains delivering a cognate TALE‐ or ArtTAL protein in leaves of wild‐type Kitaake or *sweet14‐32* edited plants. Clade‐III *SWEET* genes expression was measured by RT‐qPCR two days post‐infiltration of the *sweet14‐32* line or the control background genotype Kitaake (see legend) with bacterial strains indicated underneath the *x*‐axes. Bars represent average expression obtained from three independent RNA samples, with standard deviation. This experiment was repeated twice with similar results. Compared to the control BAI3 *talC* ^−^ strain carrying an empty vector, the BAI3 *talC* ^−^ strain expressing TalC from a plasmid (Streubel *et al*., 2013) strongly induced *SWEET14* in Kitaake but not in the *sweet14‐32* background. For the other clade‐III SWEETs, we observed a strong induction by their cognate TALE (PthXo1 from PXO99A [Yang *et al*., 2006;]) or ArtTAL (Streubel *et al*., 2013) relative to the negative control, irrespective of the plant genetic background.

**Table 1** Detailed information on the edited lines and mutation events selected and studied for AvrXa7 and Tal5 EBEs. y, yes; n, no; nd, not determined.

**Table 2** Detailed information on the edited lines and mutation events selected and studied for TalC EBE. y, yes; n, no; nd, not determined.
