# Supplementary figures and images for: Targeted promoter editing for rice resistance to Xanthomonas oryzae pv. oryzae reveals differential activities for SWEET14‐inducing TAL effectors
Source: Plant Biotechnol J. 2016 Dec 17;15(3):306–17. doi: 10.1111/pbi.12613 (PMC5316920; doi:10.1111/pbi.12613)

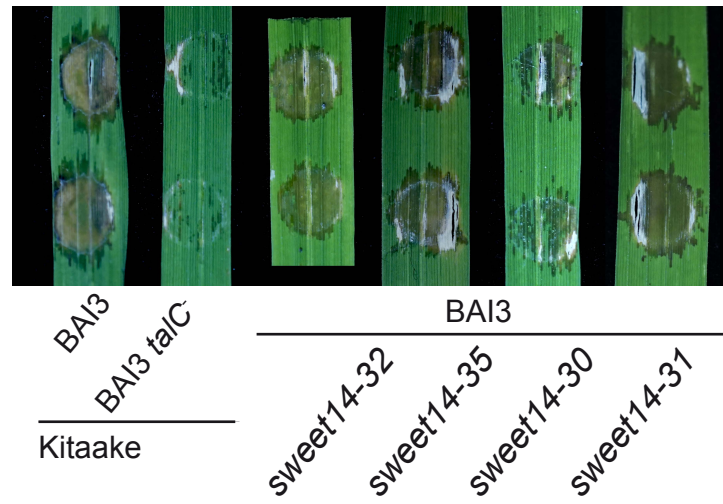

**Supplementary Figure 1**

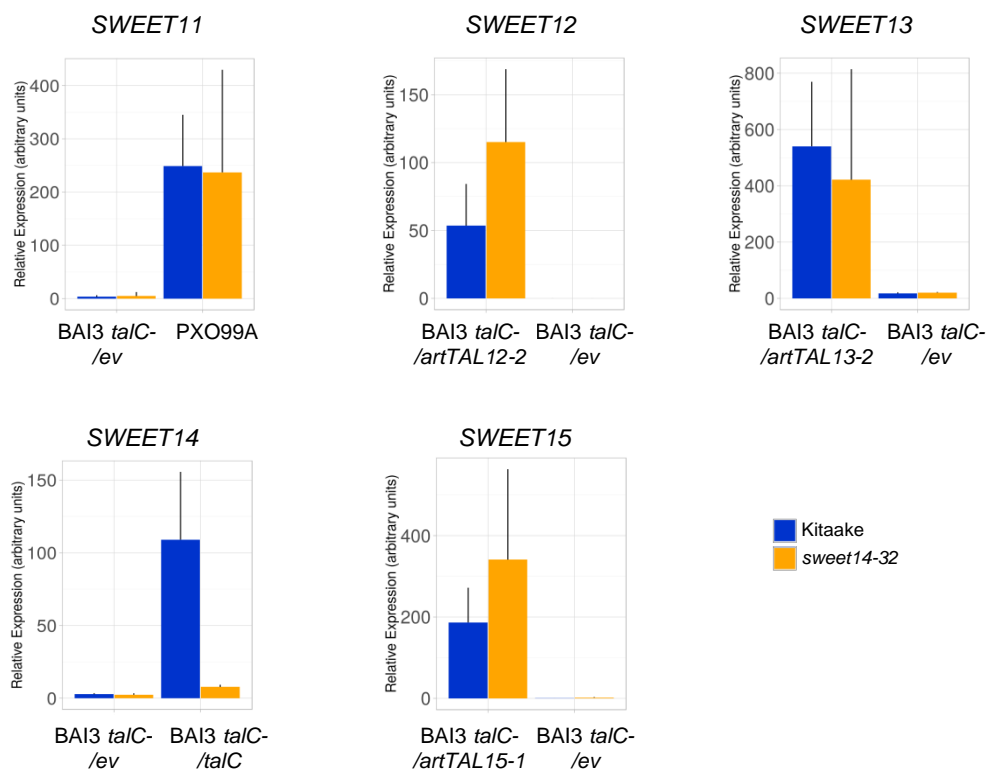

**Supplementary Figure 2**

Supplement: Supplementary file 1 — Figure 1 Water‐soaking symptoms obtained for the four TalC EBE‐edited lines studied at five days after infiltration with BAI3 wild‐type bacteria. Figure 2 Clade‐III SWEET genes upregulation in response to bacterial strains delivering a cognate TALE‐ or ArtTAL protein in leaves of wild‐type Kitaake or sweet14‐32 edited plants. Clade‐III SWEET genes expression was measured by RT‐qPCR two days post‐infiltration of the sweet14‐32 line or the control background genotype Kitaake (see legend) with bacterial strains indicated underneath the x‐axes. Bars represent average expression obtained from three independent RNA samples, with standard deviation. This experiment was repeated twice with similar results. Compared to the control BAI3 talC − strain carrying an empty vector, the BAI3 talC − strain expressing TalC from a plasmid (Streubel et al., 2013) strongly induced SWEET14 in Kitaake but not in the sweet14‐32 background. For the other clade‐III SWEETs, we observed a strong induction by their cognate TALE (PthXo1 from PXO99A [Yang et al., 2006;]) or ArtTAL (Streubel et al., 2013) relative to the negative control, irrespective of the plant genetic background. [file PBI-15-306-s002.pdf]
